# Phenazone Poisoning

**Published:** 1903-08-01

**Authors:** 


### PHENAZONE POISONING.

Toxic symptons after the administration of phenazone are not so often recorded now as they were some years ago. As, however, the drug is included in some proprietary articles which are said to have a large sale, and is moreover not infrequently dispensed by chemists in the form of tablets, it may not be out of place to notice briefly the symptoms which may occur after small or moderate doses, owing to a peculiar idiosyncracy which exists in certain persons. A fairly typical case recently came under the writer's care, of which a short description will first be given. The patient, an anæmic young woman, one evening took 10 grains of phenazone obtained from a chemist as a remedy for headache. In about 15 minutes she began to feel ill, shivering and perspiring freely. When seen a short time after she was found to have considerable œdema of the face, the eyes being almost closed, and an erythematous patchy rash on the neck and arms, intermingled with small white papules, and most marked on the extensor surfaces. There was much itching, a weak rapid pulse, and a temperature in the mouth of 95° F. During the night she vomited once. Next day the rash had gone ; the urine was examined and found to be highly acid, with a thick deposit of urates but otherwise normal. The pulse continued weak and the temperature subnormal till the second day. The œdema did not subside till the fourth day, during which time the patient felt weak and ill. The writer has collected 23 cases from the English and American literature, 15 in females, six in males, and two in children. In 18 cases a single dose had been given. In adults this varied from four to 18 grains, and the children had seven and a half grains and three-quarters of a grain respectively. The toxic symptoms in most cases appeared in 10 or 15 minutes, but in three were noticed immediately. The most usual initial symptom was a rash (seven cases), and next in frequency came internal sensations described as choking, burning, or suffocating (five cases). In the 23 cases the following symptoms were noticed. Burning or swelling of the throat eight cases, œdema of the face six cases, collapse and vomiting five cases each, coryza and cyanosis three cases each, sweating two cases. More unusual symptoms were hæmaturia, in one case after 60 grains taken during 30 hours, amaurosis in one case after 10 grains, loss of power in the left side followed by temporary cyanosis and "pins and needles" in the left arm in one case after 10 grains, and severe mental symptoms in one case, but here the dosage was high, 75 grains having been taken each day for two days. In six cases the pulse is stated to have been weak and rapid, in one intermittent, and in one full and rapid. In only one case, after about 15 grains, was recovery at all retarded, and here much weakness and occasional rashes were noted for three weeks. The rash varied in character. It was absent in seven cases, in seven it was urticarial, in four erythematous, and in three a combination of urticaria and erythema. In one case it was morbilliform, and in one occurred as a herpetic eruption on the face and genitals. Itching was absent in two of the erythematous cases. In this series the extensor surfaces do not seem to have been especially involved; in three cases, indeed, the inner surfaces of the thighs and lower part of the abdomen was the seat of the eruption. In nearly all the rash was transitory. In most cases similar doses produced the same effect every time; in some smaller doses could be borne and occasionally a patient who had previously taken the drug with impunity unexpectedly developed toxic symptoms. In the majority serious disease was absent when the drug was taken. Stimulants, such as whisky or sal volatile, were given with the phenazone in a few cases, but failed to prevent the unpleasant results. The treatment is in all cases symtomatic, but a purgative is generally indicated to assist in the elimination of the poison.

